# Effect of nanoparticles concentration on electromagnetic-assisted oil recovery using ZnO nanofluids

**DOI:** 10.1371/journal.pone.0244738

**Published:** 2020-12-31

**Authors:** Muhammad Adil, Keanchuan Lee, Hasnah Mohd Zaid, M. Fadhllullah A. Shukur, Takaaki Manaka

**Affiliations:** 1 Department of Fundamental and Applied Sciences, Universiti Teknologi PETRONAS, Bandar Seri Iskandar, Tronoh, Perak, Malaysia; 2 Department of Electrical and Electronic Engineering, Tokyo Institute of Technology, Meguro-ku, Tokyo, Japan; Brandeis University, UNITED STATES

## Abstract

Utilization of metal-oxide nanoparticles (NPs) in enhanced oil recovery (EOR) has generated substantial recent research interest in this area. Among these NPs, zinc oxide nanoparticles (ZnO-NPs) have demonstrated promising results in improving oil recovery due to their prominent thermal properties. These nanoparticles can also be polarized by electromagnetic (EM) field, which offers a unique Nano-EOR approach called EM-assisted Nano-EOR. However, the impact of NPs concentrations on oil recovery mechanism under EM field has not been well established. For this purpose, ZnO nanofluids (ZnO-NFs) of two different particle sizes (55.7 and 117.1 nm) were formed by dispersing NPs between 0.01 wt.% to 0.1 wt.% in a basefluid of sodium dodecylbenzenesulfonate (SDBS) and NaCl to study their effect on oil recovery mechanism under the electromagnetic field. This mechanism involved parameters, including mobility ratio, interfacial tension (IFT) and wettability. The displacement tests were conducted in water-wet sandpacks at 95˚C, by employing crude oil from Tapis. Three tertiary recovery scenarios have been performed, including (i) SDBS surfactant flooding as a reference, (ii) ZnO-NFs flooding, and (iii) EM-assisted ZnO-NFs flooding. Compare with incremental oil recovery from surfactant flooding (2.1% original oil in place/OOIP), nanofluid flooding reaches up to 10.2% of OOIP at optimal 0.1 wt.% ZnO (55.7 nm). Meanwhile, EM-assisted nanofluid flooding at 0.1 wt.% ZnO provides a maximum oil recovery of 10.39% and 13.08% of OOIP under EM frequency of 18.8 and 167 MHz, respectively. By assessing the IFT/contact angle and mobility ratio, the optimal NPs concentration to achieve a favorable ER effect and interfacial disturbance is determined, correlated to smaller hydrodynamic-sized nanoparticles that cause strong electrostatic repulsion between particles.

## Introduction

In recent years, nanoparticles (NPs) have employed as a promising means of improving the reservoir characteristics and increasing oil recovery, resulting in the term of Nano-EOR [1–3]. Nano-EOR has a few advantages compared to conventional EOR techniques. The small particle sizes of less than 100 nm can move easily through a reservoir rock's pore throats and distribut to the pore network locations where they can provide an efficient and noticeable difference in various ways. The NPs deposition could alter the governing properties of the displacement fluid, including viscosity [4,5], interfacial tension [6,7], dielectric properties [8]; alter the rock permeability [9]; or change the wettability of the rock surface [6,10]. Therefore, the size-dependent properties (such as optical, electrical, magnetic, interfacial, and thermo-physical properties) of NPs are crucial to aim locations challenging for conventional methods to access, such as sensitive downhole sensors [1,11,12].

Nanotechnology’s rapid progress has generated different forms of nanoparticles, such as metal-oxides for multi-purpose applications in various fields. At present, nanometals play a key role in multiple areas of physics, chemistry, and materials science [13]. Metal-oxide based NFs for heat transfer and thermal conductivity purposes are often employed [14–16]. The most commonly used metal oxide NPs are Al_2_O_3_, ZnO, TiO_2_, Fe_2_O_3_, MgO, CeO_2_, and ZrO_2_, where each of them possesses distinctive physical and chemical properties [13]. In the case of EOR, such nanoparticles designed to facilitate modification of wettability, reduction of viscosity ratio, stability of foam or emulsion and reduction of IFT–in certain cases employing electric or magnetic field. Ogolo et al. [17] studied eight NPs, including ZnO, Al_2_O_3_, MgO, Fe_2_O_3_, ZrO_2_, NiO, SnO_2_, and SiO_2_ and observed that alumina NPs of an average size of 40 nm produced a higher tertiary oil recovery (12.5%) through reduction of viscosity ratio compared to other nanometals, when dispersed in brine. In a two-phase coreflooding process, Hendraningrat et al. [18] investigate the potential of Al_2_O_3_ and TiO_2_ NPs, and demonstrated an increased oil production as a result of better NPs adsorption into the pore surface. Once metal oxides introduced to the brine, it reduces contact angle from 54˚ to 21˚ which means metal oxides have changed the quartz plate to be more strongly water-wet. The highest reduction in contact angle was achieved from TiO_2_, corresponding to the greater oil recovery. Joonaki and Ghanaatian [19] studied the impact of nano-oxides (aluminum-, iron-, and silicon-oxide) on the IFT and noticed that increasing NPs concentration decreased the IFT. Zargartalebietal et al. [20] also determined the impact of fumed SiO_2_ NPs on the effectiveness of surfactant in reducing IFT. They found that the introduction of NPs at lower surfactant concentrations reduced the IFT. However, IFT increased at higher surfactant concentrations upon the addition of NPs. Similar observations were noticed for ZrO_2_ NPs by Esmaeilzadeh et al. [21]. Hendraningrat et al. [22,23] suggested that the IFT between synthetic oil and NFs is sensitive to nanofluid concentration, and demonstrated that increasing NPs concentration from 0.01 ‒ 0.05 wt.% reduced the IFT from 9.3 mNm^-1^ to 5.2 mNm^-1^. In another study, Hendraningrat and Torseater [18] have identified a process to improve oil recovery by modifying the wettability of Al_2_O_3_, SiO_2_, and TiO_2_ NPs. They found that the contact angle changed towards water-wet state as particle size decreased, leading to increased oil recovery. Ehtesabietal et al. [24] employed TiO_2_ NPs in core flooding experiments using sandstone core and recovered up to 31% additional oil recovery using the 0.01% TiO_2_ NPs. However, the TiO_2_ NPs concentration change to 1% did not achieve any noticeable change in the recovery factor. This suggests that an optimal NPs concentration is crucial to achieve a maximum oil recovery, and higher NPs concentrations have no substantial impact on the recovery factor, rather an increase in the cost only.

These studies have proven that the oil recovery improved by using ZnO-NPs under the application of EM field. However, the impact of NPs concentration on oil recovery under EM field is still lacking, since the choice of nanoparticles' material, size, concentration, and stability in high salinities greatly influenced the displacement fluid properties. Therefore, to further understand the impact of NPs concentration on EM-assisted oil recovery, this paper has employed nanofluids with varying concentrations of ZnO-NPs, offering different particle sizes as well as dielectric behavior at a given EM frequency. The dielectric properties are crucial to render NPs as surface-active agents that polarized under an external EM field, resulting in the formulation of a unique oil recovery mechanism. The current investigation is an extension study of our previous research [25], which aims to demonstrate the effect of ZnO-NFs with varying concentration of NPs and their average hydrodynamic size on oil recovery corresponding to the applied EM frequency.

## Materials and methods

### Materials

The as-prepared ZnO-NPs, used for this study, were prepared earlier by employing the sol-gel auto-combustion approach [26,27], and calcined at 500 ˚C and 800 ˚C to produce the final product; denoted as ZnO@500 and ZnO@800, respectively [28]. Table 1 lists the average sizes of these NPs obtained from transmission electron microscopy (TEM) & X-ray diffraction (XRD) corresponding to their calcination temperatures. At the same time, their TEM images and XRD pattern can be seen in S1–S4 Figs. The analytical grade of sodium dodecylbenzenesulfonate (SDBS, Sigma Aldrich) was used as a stabilizer without further purification. For oil recovery tests, crude oil sourced from Tapis, Malaysia (geographical coordinates of 05˚ 310 44.85 @ N, 104˚ 57 0 20.77 @ E) has been used as an oil phase, purchased from Petronas Penapisan (Melaka) Sdn Bhd, Malaysia. On the other hand, sodium chloride (NaCl, Fisher Scientific) was used as a base aqueous phase to prepare brine in deionized water (with σ = 18MΩ) at 3 wt.% (≈ seawater concentration). The 3 wt.% NaCl solution was also employed as a saturation and injection fluid, since brine similar to seawater is produced from oil reservoirs and readily accessible in offshore fields.

**Table 1:** ZnO-NPs sizes at different calcination temperatures.

Nano Sample	Nanoparticle Size (nm)	
	Transmission electron microscopy (TEM)	X-ray diffraction (XRD)^a^
ZnO@500	55.7	43.4
ZnO@800	117.1	47.3

^a^ Crystallite size

### Nanofluid preparation

The ZnO nanoparticles with concentration between 0.01–0.1 wt.% dispersed in the brine to prepare the NFs by stirring magnetically for 1 hour, which acts as a basefluid. Then, an optimal concentration of 0.025 wt.% SDBS was mixed as a stabilizer to the NFs. This optimal concentration of SDBS was determined by using the critical micelle concentration (CMC) method [29]. The pH of NFs was adjusted to an optimal value of 2 with HCl solution using a precise pH meter (Mettler Toledo, FE20-Basic) before they were stirred in an ultrasonic bath for an optimum period of 60 min [30] to achieve the required nanofluid concentrations. A vibrating-tube densimeter DMA 5000 (Anton Paar) was used to measure the density of these NFs, having an accuracy of 5 × 10^‒6^ g/cm^3^ and a repeatability of 1 × 10^‒6^ g/cm^3^. Table 2 demonstrates the properties of brine, crude oil, and varying concentrations of ZnO-NFs measured at ambient condition.

**Table 2:** Fluid properties under room temperature.

Fluid	Concentration (wt.%)	Properties	
		Density (g.cm^-3^)	Viscosity @ 100 rpm (cP)
Crude oil	‒	0.8021	7.50
Brine (NaCl)	3	1.0197	1.01
SDBS	0.025	1.0194	1.02
ZnO@500	0.1	1.0204	1.05
	0.05	1.0198	1.02
	0.01	1.0178	1.02
ZnO@800	0.1	1.0205	1.07
	0.05	1.0199	1.03
	0.01	1.0194	1.02

### IFT and wettability measurements

**Fig 1:**
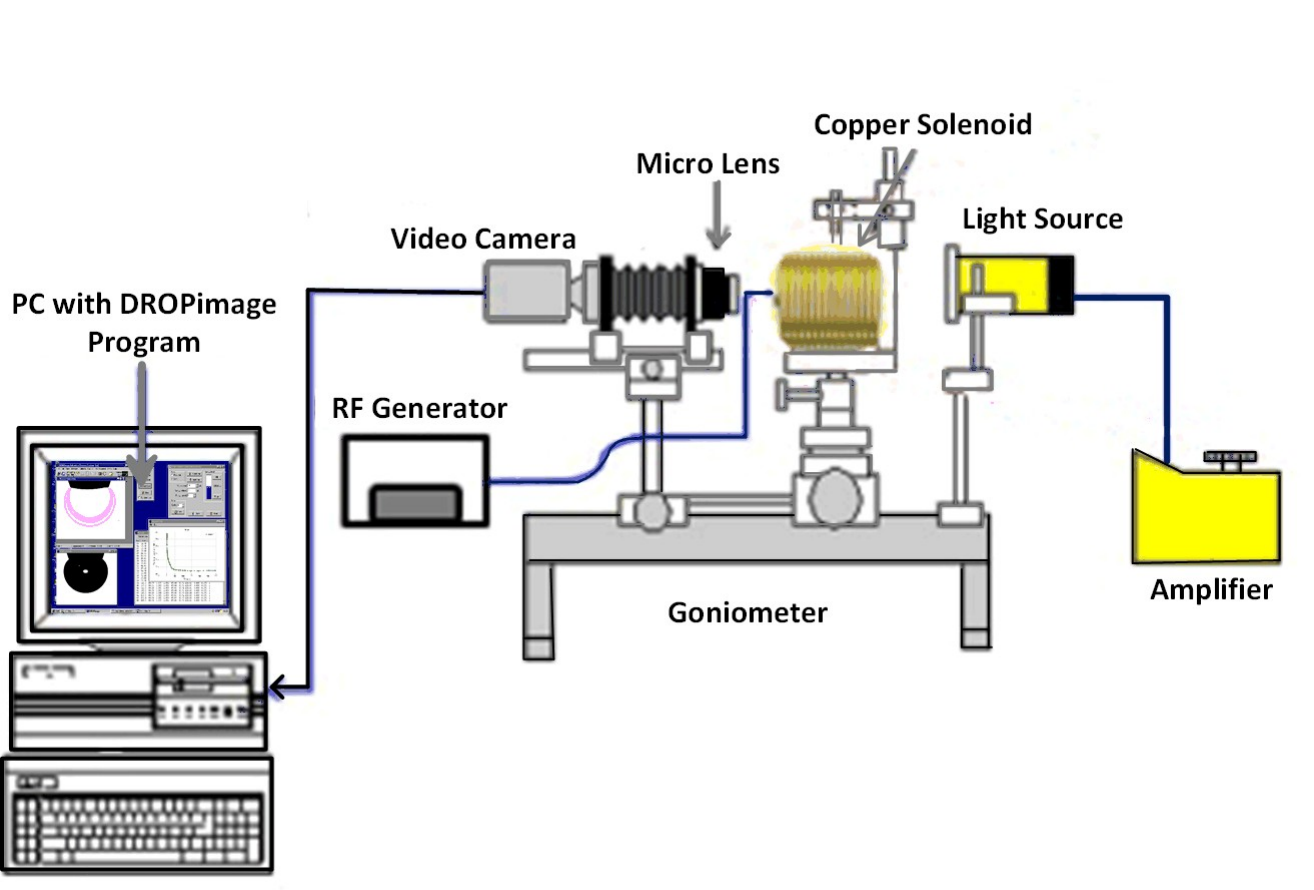
Schematic of goniometer attached with custom-made solenoid coil for the measurement of IFT and contact angle of ZnO nanofluids under electromagnetic field.

IFT between oil/brine and oil/ZnO-NFs and the three-phase contact angle between solid-ZnO NFs-oil were measured using the classic method of sessile drop-shape analysis. These measurements were performed under ambient temperature and pressure using a Goniometer (Model 260, Ramé-hart). The measurement setup consists of three major components: (i) a glass plate as a solid surface; (ii) Tapis crude oil as an oil phase; and (iii) ZnO-NFs at varying NPs concentration as an aqueous phase. Fig 1 (prepared in Microsoft Visio) depicted the setup with a glass cell container, along with a fabricated solenoid coil, situated between the source of light and the camera. The solenoid of a fixed diameter (7 cm), because of the limitation of sample size, was attached to the radio frequency (RF) generator (33500B, Agilent) to generate an electromagnetic field at a lower frequency of 18.8 MHz. Air as an EM propagation medium was used, limiting the use of higher frequency of 167 MHz; leading to a significant loss of EM strength.

**Fig 2:**
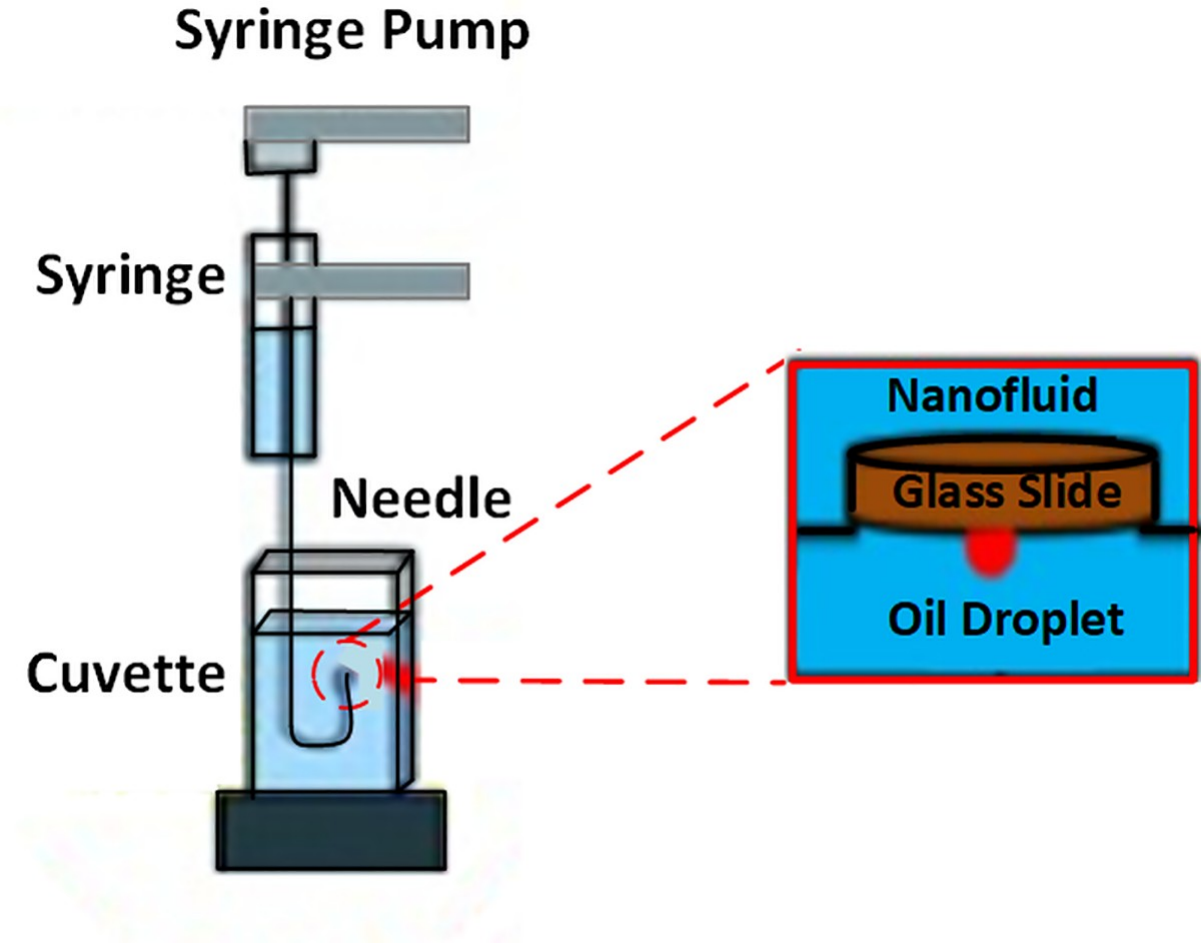
Measurement setup for a sessile-drop shape analysis comprises a glass slide, crude oil droplet, and ZnO-NFs as a solid base, oil phase, and aqueous phase, respectively.

The nano-sample container surrounded by a solenoid coil is separately depicted in Fig 2, with a glass surface representing sandstone on top of the container. An inverted syringe was employed to position a small drop of crude oil (24 ± 0.2 μL) under the glass plate. Since oil density is lower than ZnO-NFs density, it was important to place the oil drop under the plate. The camera was manually adjusted to obtain a focused and magnified image of the drop and the surface, as seen on the connected computer. Once the drop-shape profile reached equilibrium, indicating a steady IFT, the camera captured the droplet images. DROPimage software then used the side-view profile to measure the IFT and 3-phase contact angle using an image analysis approach. These images were also incorporated in the figures prepared using Origin 9 from OriginLab, depiciting IFT and contact angle results.

### Sandpack flooding

**Fig 3:**
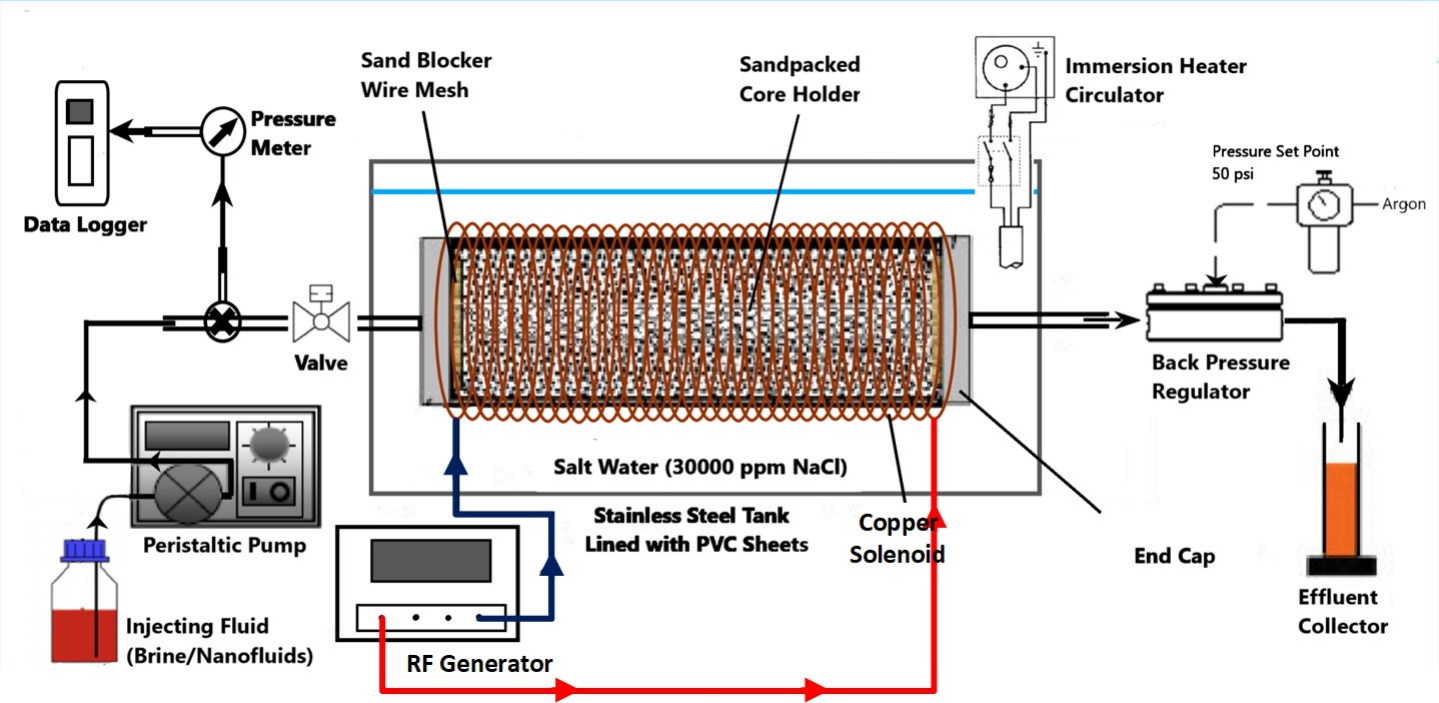
Schematic of the nano-ZnO flooding setup in the presence of EM field, produced by a copper solenoid surrounding the sandpack holder saturated with crude oil.

The acrylic core holder was used as a sandpack, having a diameter and length of 4.6 cm and 30 cm, respectively. For each displacement test, fresh quartz sand (300–425 μm mesh size) was packed to achieve the same initial wettability status and then fully saturated with brine at a 50 psi back-pressure. The porosity and permeability of sandpacks were determined to be in a range of 35%– 39% and 267–303 mD, respectively. The setup of two-phase displacement test is illustrated in Fig 3 using Microsoft Visio. In the case of EM-assisted flooding, the sandpack was placed inside a solenoid coil. These solenoid coils were specifically tailored to generate EM field under salt water at a scale down frequency of 167 and 18.8 MHz (corresponding to 1000 and 3000 m of well spacing). All the production graphs were prepared using Origin 9 from OriginLab.

A brief procedure of sandpack flooding is summarized here, while the details can be seen in our previous study [25]. The brine-saturated sandpack was injected with crude oil at 1 cm^3^/min, which lasted until no further water recovered to determine the initial water saturation (*S*_*wi*_*−*Eq 1). The injection rate of 1 cm^3^/min corresponds to 2.83 ft/day and a shear rate of 10 s^-1^, which is consistent with typical shear rates (0.01 ‒ 10 s^-1^) in most formations [31].

$$S_{wi}=\left(\frac{PV-OOIP}{PV}\right)\times 100$$ (1)

where *PV* is a pore volume, described as the empty volume of the sandpack holder, calculated by determining the brine volume required to saturate the sandpack.

As a secondary recovery process, brine (denoted as WF) was pumped at 1 cm^3^/min to recover the oil, while the system’s temperature and pressure were kept at 95 ˚C and 50 psi, respectively. The average oil production after WF varies from 54.7% to 57% of OOIP, while the residual oil saturation after water flooding (*S*_*or1*_) recorded between 42.9% and 45.2% of PV. The oil displaced after primary flooding (*V*_*orwf*_, cm^3^) was determined volumetrically, while *S*_*or1*_ was estimated using:

$$S_{or1}=\left(\frac{OOIP-V_{orwf}}{OOIP}\right)\times 100$$ (2)

The injection then proceeded at an identical injection rate (1 cm^3^/min) and pore volume for ZnO-NFs as a tertiary recovery process without and with EM field, denoted as NF and EMNF, respectively. The output from NFs flooding was collected correspond to the oil recovered (*V*_*ornf*_, cm^3^), and the residual oil saturation after nano-EOR (*S*_*or2*_) as:

$$S_{or2}=\left(\frac{OOIP-V_{orwf}-V_{ornf}}{OOIP}\right)\times 100$$ (3)

## Results and discussion

In this study, three types of tertiary flooding have been performed. In the first type, the aqueous solution of 0.025 wt.% SDBS was injected as a base case, while the tertiary recovery for the second type used various weight percentages of both ZnO@500 and ZnO@800 NPs. In the last type, these ZnO nanofluids have been flooded along with electromagnetic field after primary flooding to monitor the EM-assisted EOR. In these displacement experiments, the impact of ZnO-NFs with varying concentration of nanoparticles and their corresponding hydrodynamic sizes on the conventional and EM-assisted tertiary recovery were investigated. The detailed experimental results comprising of oil production profiles can be seen in S1–S4 Figs.

In the case of a given temperature, the displacement efficiency (*E*_*D*_, %) of tertiary recovery process, reflecting the fingering due to the loss of mobility control in the fluid flow has also been assessed [32]. The *E*_*D*_ is calculated using the formula as follows:

$$E_{D}=\left[1-(\frac{S_{or2}}{S_{or1}})\right]\times 100$$ (4)

### Surfactant flooding

**Fig 4:**
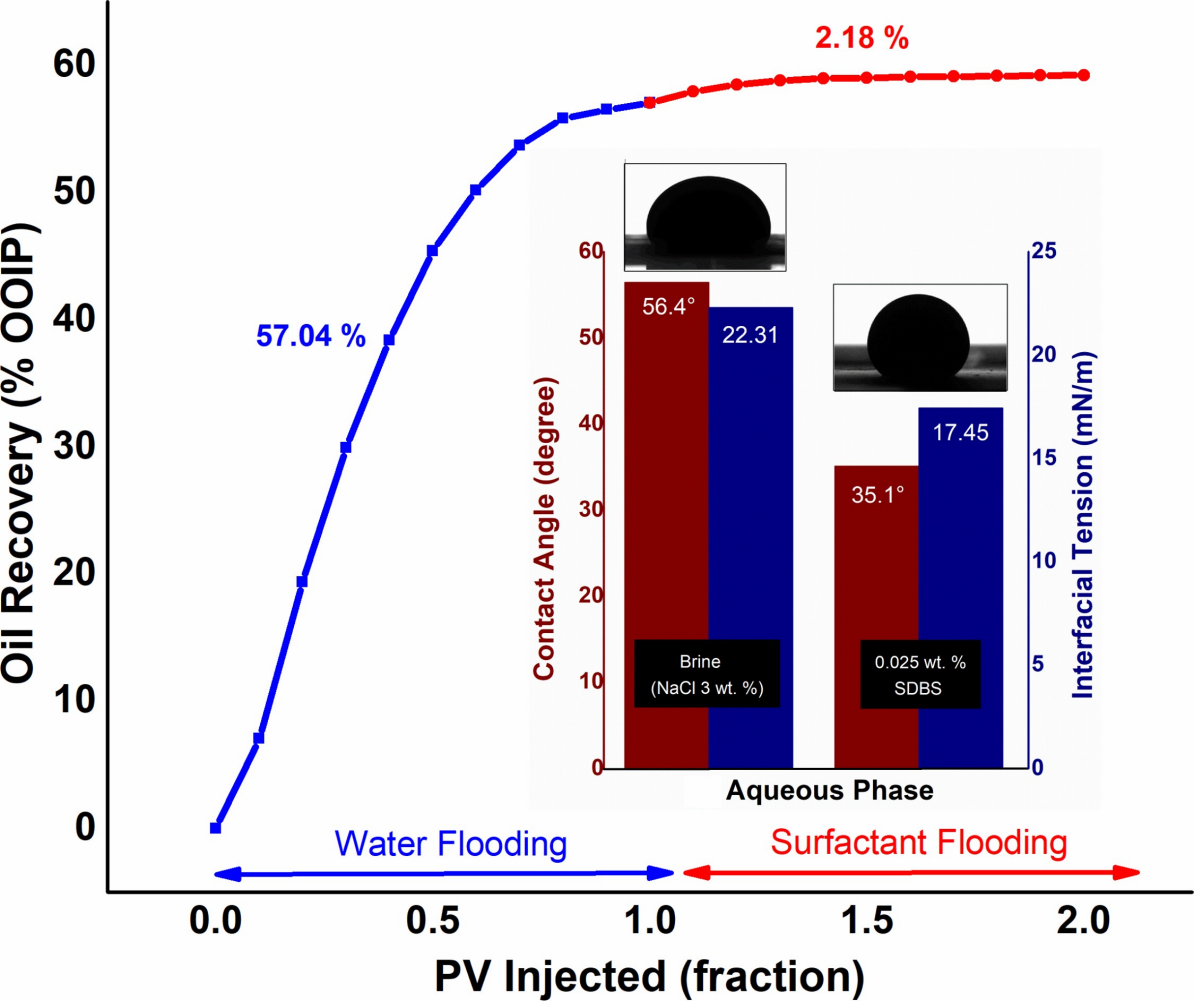
Production performance of 0.025 wt.% SDBS flooding, along with IFT and contact angle values against crude oil.

In the first scheme, after waterflooding, 1 PV of 0.025 wt.% SDBS solution injected as a base study for EOR. As depicted in Fig 4, the surfactant flooding provides a negligible oil recovery improvement of 2.1% of OOIP, after primary flooding. Oil output declined after the injection of 0.5 PV of SDBS, while the plateau area on the curve observed after 0.7 PV and beyond suggests that no additional oil could extract. Although SDBS decreases the IFT between oil and surfactant solution, as well as causes change in wettability at ambient condition (see Fig 4). However, SDBS continues to degrade at high temperatures [33], which decreases the interaction between the crude oil and surfactant with a viscosity ratio of 7.56. Consequently, reduced the oil recovery.

### Conventional nanofluid flooding

**Fig 5:**
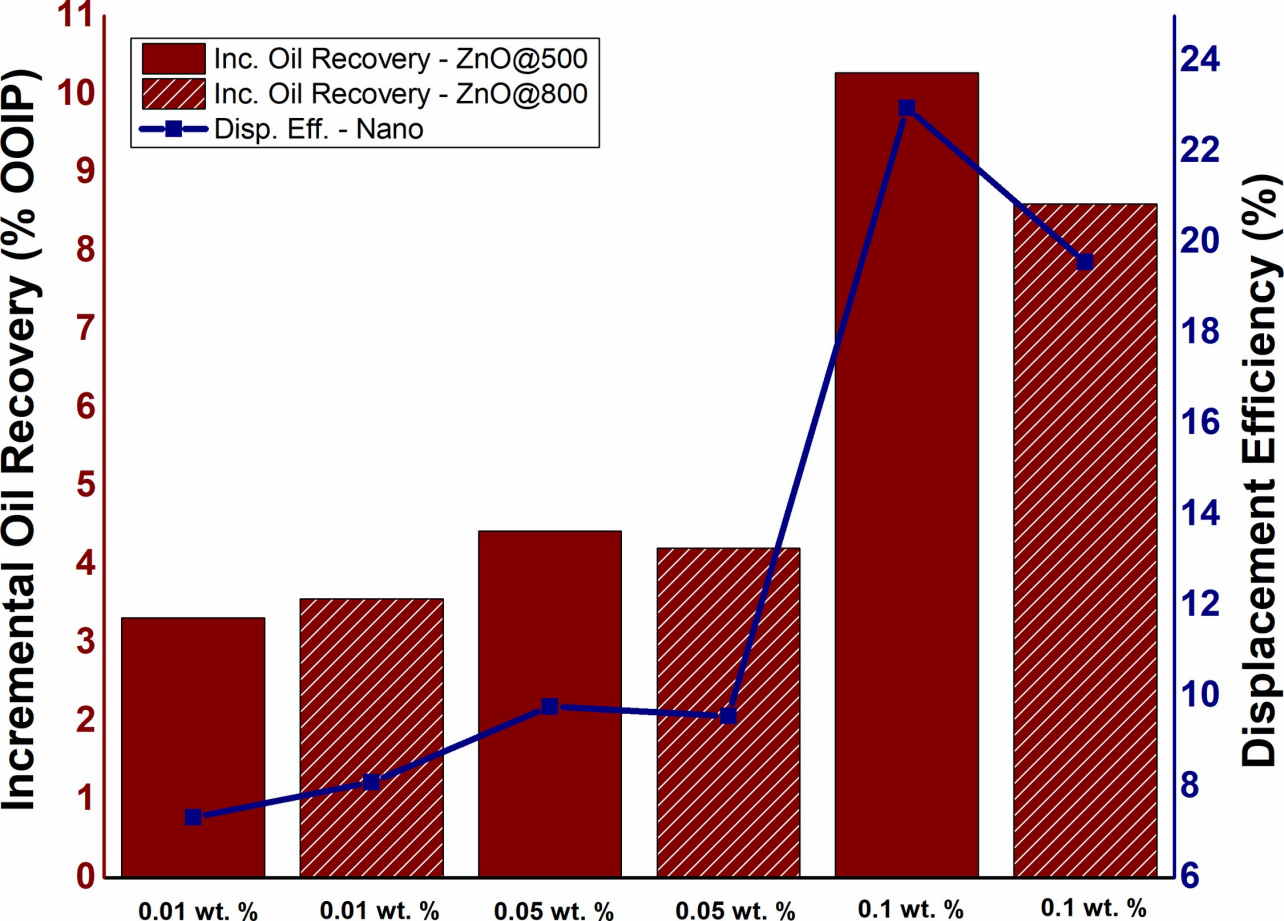
The influence of NPs concentration on oil production and displacement efficiency of ZnO@500 and ZnO@800 NFs under conventional nanofluid flooding.

To determine the impact of NPs concentration on tertiary oil recovery along with displacement efficiency, ZnO-NFs with various concentrations (0.01 wt.%, 0.05 wt.%, and 0.1 wt.%) were continuously injected (1 PV). As depicted in Fig 5, the oil production increased from 3.31 to 10.27% OOIP as the NPs concentration increased from 0.01 to 0.1 wt.%. While the highest oil recovery of 10.2% OOIP observed for 0.1 wt.% ZnO@500 NPs, which is significantly higher than SDBS alone. However, the NFs required a certain period during nanofluid flooding to displace incremental oil, likely caused by physicochemical interactions between the ZnO nanoparticles and the sandpack. A similar trend observed for displacement efficiency with the highest efficiency achieved by 0.1 wt.% ZnO-NFs. These findings are in accordance with Hendraningrat et al. [23], depicted that oil production improves with the increase in NPs concentration.

**Fig 6:**
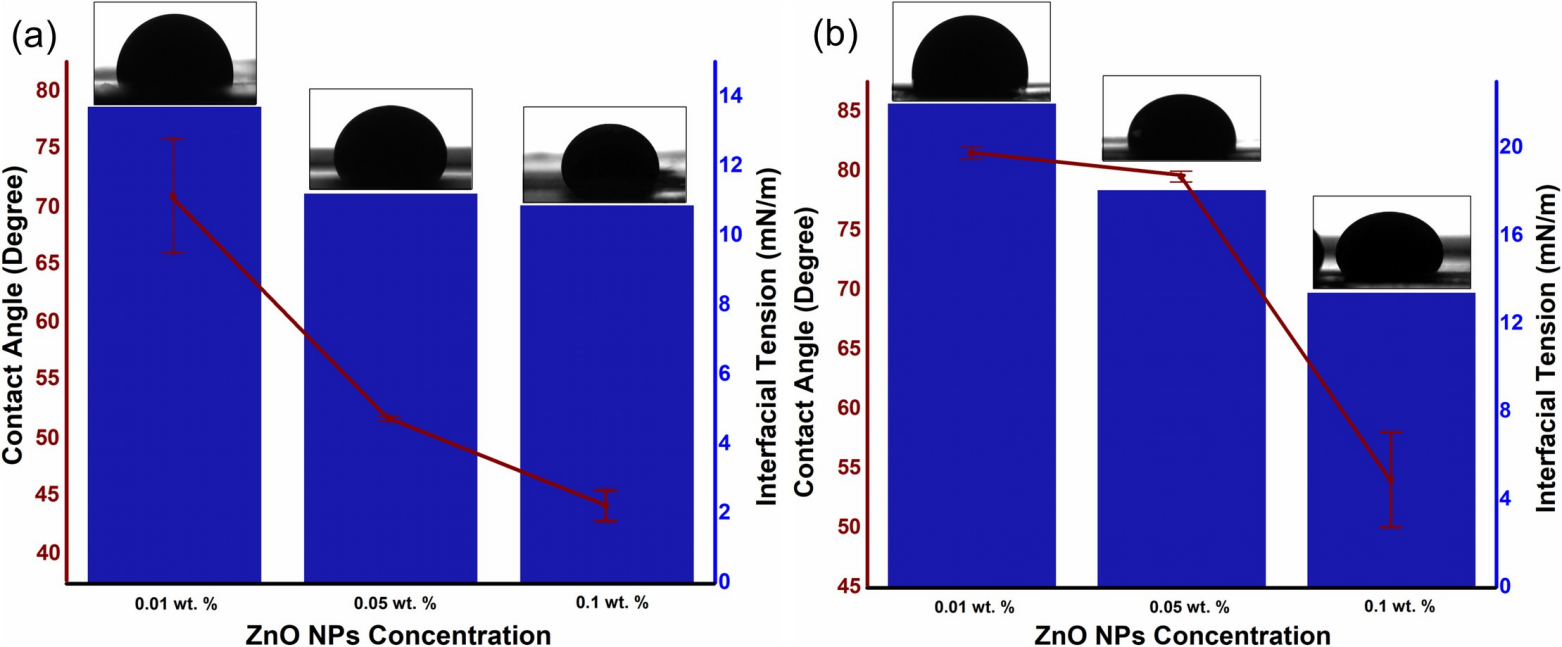
Effect of NPs concentration on IFT as well as contact angle (with images) of (a) ZnO@500 NFs and (b) ZnO@800 NFs, under ambient conditions.

The dependence of additional oil recovery on nanofluid concentration is in accordance with the IFT and contact angle, as well as the viscosity ratio. As shown in Fig 6, the changes in IFT and contact angle is also a function of NPs concentration, where the IFT and contact angle reduces with the increase in particle concentration. Moreover, in the presence of 0.025 wt.% SDBS, the hydrodynamic size of ZnO-NFs decreases with the increase in NPs concentration as reported in our previous work [30,34] (see Table 3). Note that the SDBS concentration is only optimized for 0.1 wt.% ZnO-NPs. Therefore, the large surface-to-volume ratio of smaller hydrodynamic-sized nanoparticle causes an increase in surface free-energy, which contributes to a decline in IFT. This decrease of IFT due to the interaction of NPs with the interface between crude oil and SDBS could be the reason for difference in tension gradient. Binks [35] also noticed the changes in the IFT as a function of NPs concentration. He proposed that the difference in the IFT values of the various concentrations of the NFs attributed to the surface energy of the NPs, which is directly related to the average particle size as suggested in Eq (5):

$$E=\pi r^{2}\gamma_{wo}(1\pm \cos\theta )$$ (5)

where *r* is the particle radius, *γ*_*wo*_ is the interfacial tension between the two fluids involved and *θ* is the contact angle with the solid surface.

**Table 3:** Hydrodynamic particle size of ZnO-NFs at a varying concentration in 0.025 wt.% of SDBS as a basefluid.

NPs concentration (wt.%)	Average hydrodynamic size (nm)	
	ZnO@500	ZnO@800
0.1	240.9	287.2
0.05	308.4	427.5
0.01	395.7	761.4

The results for the contact angle are in accordance with the IFT, where the contact angle decreases as the NPs concentration increases; rendering the glass plate more water wet. As a result, the wettability of the porous medium may be altered and will favor the aqueous phase. This reduction in water-wetness linked to the SDBS adsorption on ZnO-NPs, which allows them to remain individual in the basefluid due to the negative surface charges and provide the repulsive force with the negatively charged quartz plate [30]. The observed variations in the contact angle as a function of NPs concentration relied on the solid surface material, along with the particle size. For the identical weight percentage of both ZnO nanoparticles, smaller NPs provided significant improvement in the contact angle. Observations from the study of Vafaei et al. also showed that the smaller nanoparticles were additionally effective in reducing the sessile droplet contact angle [36].

Meanwhile, the viscosity ratio of oil to ZnO nanofluid decreased from 3.52 (0.01 wt.%) to 1.57 (0.1 wt.%), which plays a supporting role in oil recovery by diverting injected NFs into unswept areas due to the mobility ratio reduction. At high temperature, the incremental oil is produced not only because of the reduced mobility ratio, but also due to the IFT reduction at elevated temperature as the molecular interactions are weaker between the aqueous phase. Another reason is the Brownian motion intensity, which increases both with the medium’s temperature as well as decrement in its viscosity and particle size [37]. Since Brownian motions may be among the energies causing oil movement because of NPs, the force would increase. Overall, the suspension of small hydrodynamic-sized ZnO-NPs appears to be very promising at higher temperature environments, such as oil reservoirs.

### EM-assisted nanofluid flooding

**Fig 7:**
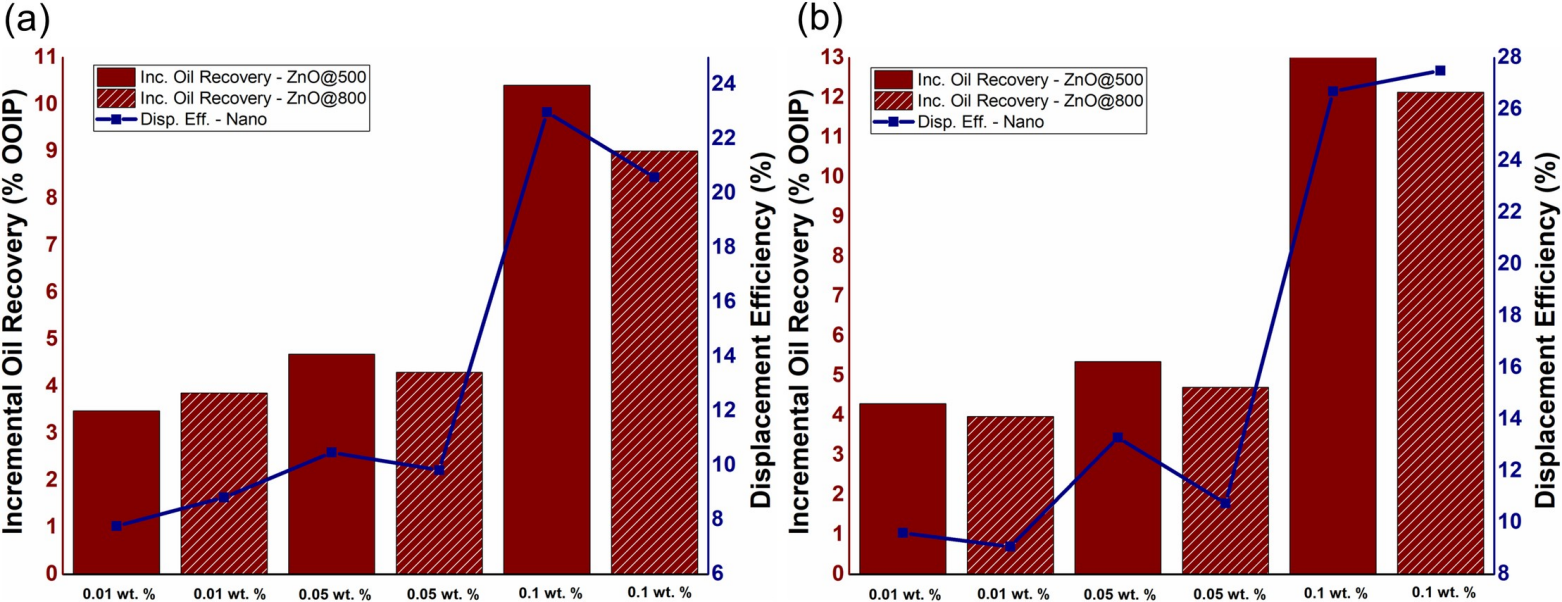
The effect of NPs concentration of ZnO@500 and ZnO@800 NFs on incremental oil production and displacement efficiency under EM-assisted nanofluid flooding at a frequency of (a) 18.8 MHz and (b) 167 MHz.

The influence of NPs concentration on incremental oil recovery, as well as displacement efficiency of EM-assisted nano-EOR flooding is depicted in Fig 7A and 7B at 18.8 & 167 MHz, respectively. The results showed that nanoparticles concentration obviously influenced incremental oil recovery due to EM-assisted Nano-EOR, by following the same displacement mechanism as discussed in Section 3.2. As in conventional Nano-EOR, the increasing NPs concentration of ZnO will also increase incremental oil recovery for EM-assisted nanofluid flooding. The highest additional oil recovery of 10.30% OOIP and 13.08% OOIP achieved at 0.1 wt.% ZnO@500 for both the applied frequencies of 18.8 and 167 MHz, respectively. While at 0.1 wt.% ZnO@800, the incremental recovery of 9% OOIP and 12.13% recorded under the EM frequencies of 18.8 and 167 MHz, respectively. Therefore, the incremental oil displacement for EM-nanofluid flooding is relatively greater than conventional nanofluid flooding. The trend also shows the displacement efficiency due to EM- assisted nano-EOR will increase as particle concentration increases.

**Fig 8:**
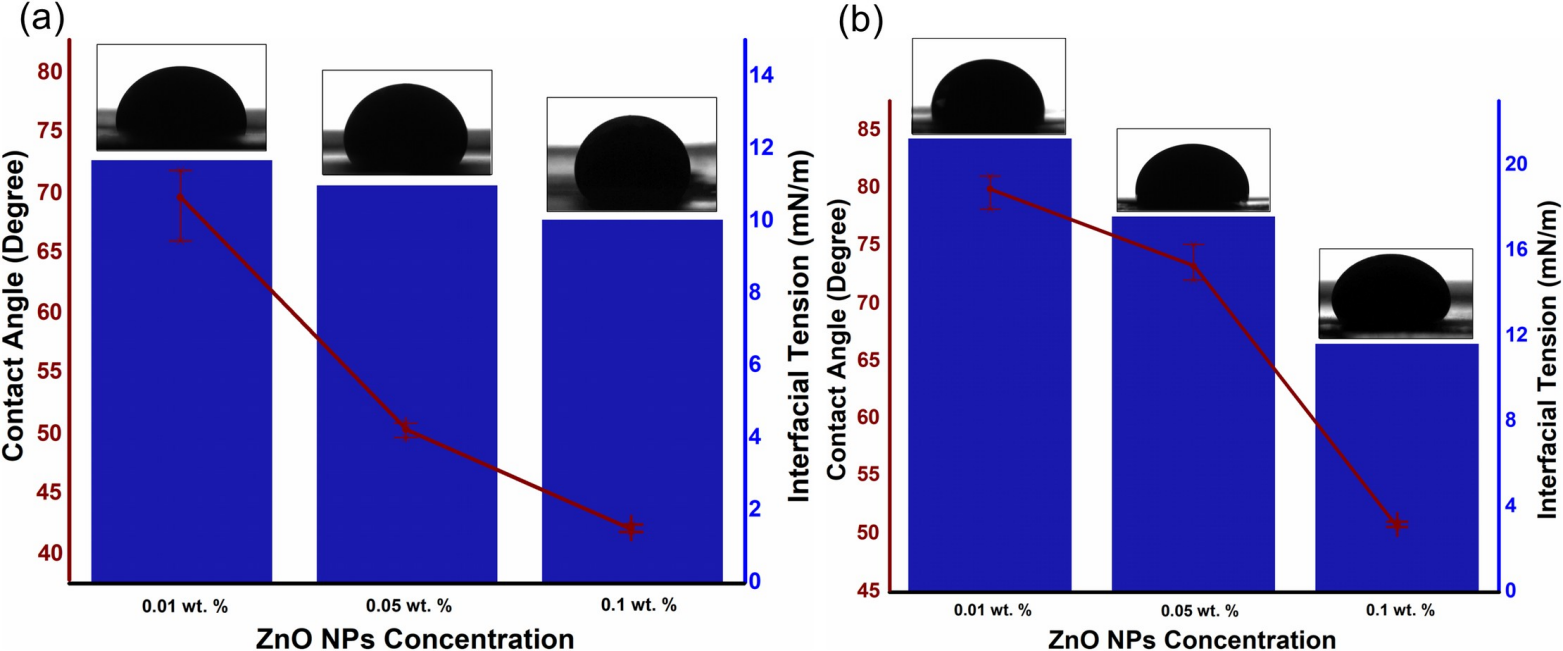
IFT and contact angle measurement of an oil droplet, under EM field, against varying concentrations of (a) ZnO@500 NFs and (b) ZnO@800 NFs, as an aqueous medium.

The influence of particle concentration on EM-assisted oil recovery linked with the IFT/contact angle of aqueous phase (as shown in Fig 8) as well as mobility, which not only decreases with the increase in particle concentration, but also exhibits additional decrement under EM field due to their unique behaviors. These behaviors include: (i) the oil droplet deformation by the polarization of attached NPs, which maximizes the surface area for the additional particle adsorption and therefore resulting in IFT reduction [38]; (ii) the increase in wettability alteration rate caused by electrowetting [38]; and (iii) the enhancement of mobility ratio caused by ER effect which improves the viscosity of ZnO-NFs [30]. The ZnO NPs, dispersed in an aqueous solution of SDBS, self- assembled at the oil/NF interface to reduce the interfacial forces generated by a single layer of NPs at the interface packed in a liquid-like form [39,40]. The NPs occupied the interface to intensify the decrement in the interfacial force and, as a result, created a disordered, jammed assembly. Upon introduction of the EM field, the oil droplet deformed, causing the surface area of the droplet to expand and unblock the assembly of NPs. This increase in the surface area allowed the aggregation of additional NPs at the oil/NF interface, which further reduced the IFT. Once the field cut off, the droplet tried to minimize the interfacial area by returning to its lowest energy shape. The layer of the assembled and jammed NPs, however, prevented any further changes in the shape of droplet, which kinetically trapped the oil droplet into a new configuration.

Meanwhile, the 3-phase contact angle of oil-NF-quartz also showed a slight decrement under the presence of the EM field, referred to as Electrowetting, first proposed by Lippmann in 1875 [41]. Electrowetting is interpreted by considering the forces applied on the fluid near the contact line, as suggested by Jones et al. [42,43]. When an EM field is applied, the free charges are drawn by the electric field near the contact line, along with the polarized dipoles of the NPs, which increases the Maxwell stress on the oil/NF interface. To counter this stress, the curvature of the oil/NF interface minimized to mitigate the Laplace pressure. Ultimately, a little apparent contact angle achieved, which depends on the polarized dipoles corresponding to the dielectric properties of the NPs. However, the extent of decrement is dependent on the dielectric behavior of NPs (especially dielectric loss) corresponding to the applied frequency (as described in our previous work [44,45]). Meanwhile, the mobility of crude oil also improved under EM field by the additional reduction in viscosity ratio from 2.61 (0.01 wt.%) to 1.09 (0.1 wt.%), compared to 3.52 (0.01 wt.%) to 1.57 (0.1 wt.%) without the presence of EM field.

## Conclusion

The additional oil recovery from EM-assisted ZnO nanofluid flooding concluded that the oil recovery mechanism is not only dependent on applied EM frequency but also relies on NPs concentration to achieve greater ER effect as well as interfacial disturbance. The minimum IFT value, 10.02 mN.m^-1^, and the smallest three-phase contact angle under EM field, 42.47˚, were achieved by the 0.1 wt.% ZnO@500 NPs, which is proportional to the additional recovery of 13.08% OOIP. This change in IFT and contact angle is related to the nanoparticles’ surface energy, which directly linked with the average hydrodynamic size (240.9 nm) of 0.1 wt.% ZnO@500 NPs. The smaller-sized particles, with good uniformity and better surface-to-volume ratio, effectively interact with the interface between the crude oil and SDBS due to a tension gradient. This, in turn, permitted the additional NPs attachment at the oil/water interface under EM field; causing IFT to reduce further. Similarly, the strong electrostatic repulsion between particles increases the surface water wetness, which allows NFs to spread along the solid surface, resulting in a decreased contact angle and improved electrowetting. On the other hand, the existence of particle agglomerates at the lower concentration of both ZnO-NPs can be responsible for poor ER effect, and ultimately low oil recovery. Overall, this study reveals that the optimal NPs concentration is as crucial as favorable dielectric properties of NPs to achieve improved oil recovery under EM field.

## Supporting information

S1 Fig Transmission electron microscopy. TEM images of ZnO nanoparticles (a, c) calcined at 500°C and 800°C respectively, and their corresponding SAED images (b, d) with inset images of lattice scale fringes.
(TIF)

S2 Fig X-ray diffraction. X-ray diffraction patterns of ZnO nanoparticles calcined at 500°C and 800°C.
(TIF)

S3 Fig Oil recovery profile for surfactant and conventional nanofluid flooding. Cumulative oil recovery as a function of injected PV for surfactant flooding of (a) 0.25 wt.% SDBS, and conventional nanofluid flooding of different NPs concentration of (b) ZnO@500 NFs and (c) ZnO@800 NFs.
(TIF)

S4 Fig Cumulative oil recovery for EM-assisted nanofluid flooding. Recovery performance of EM-assisted nanofluid flooding as a function of injected PV for different NPs concentration of (a, c) ZnO@500 NF and (b, d) ZnO@800 NF at an applied frequency of 18.8 and 167 MHz, respectively.
(TIF)
