# Supplementary figures and images for: Effect of nanoparticles concentration on electromagnetic-assisted oil recovery using ZnO nanofluids
Source: PLoS One. 2020 Dec 31;15(12):e0244738. doi: 10.1371/journal.pone.0244738 (PMC7774934; doi:10.1371/journal.pone.0244738)

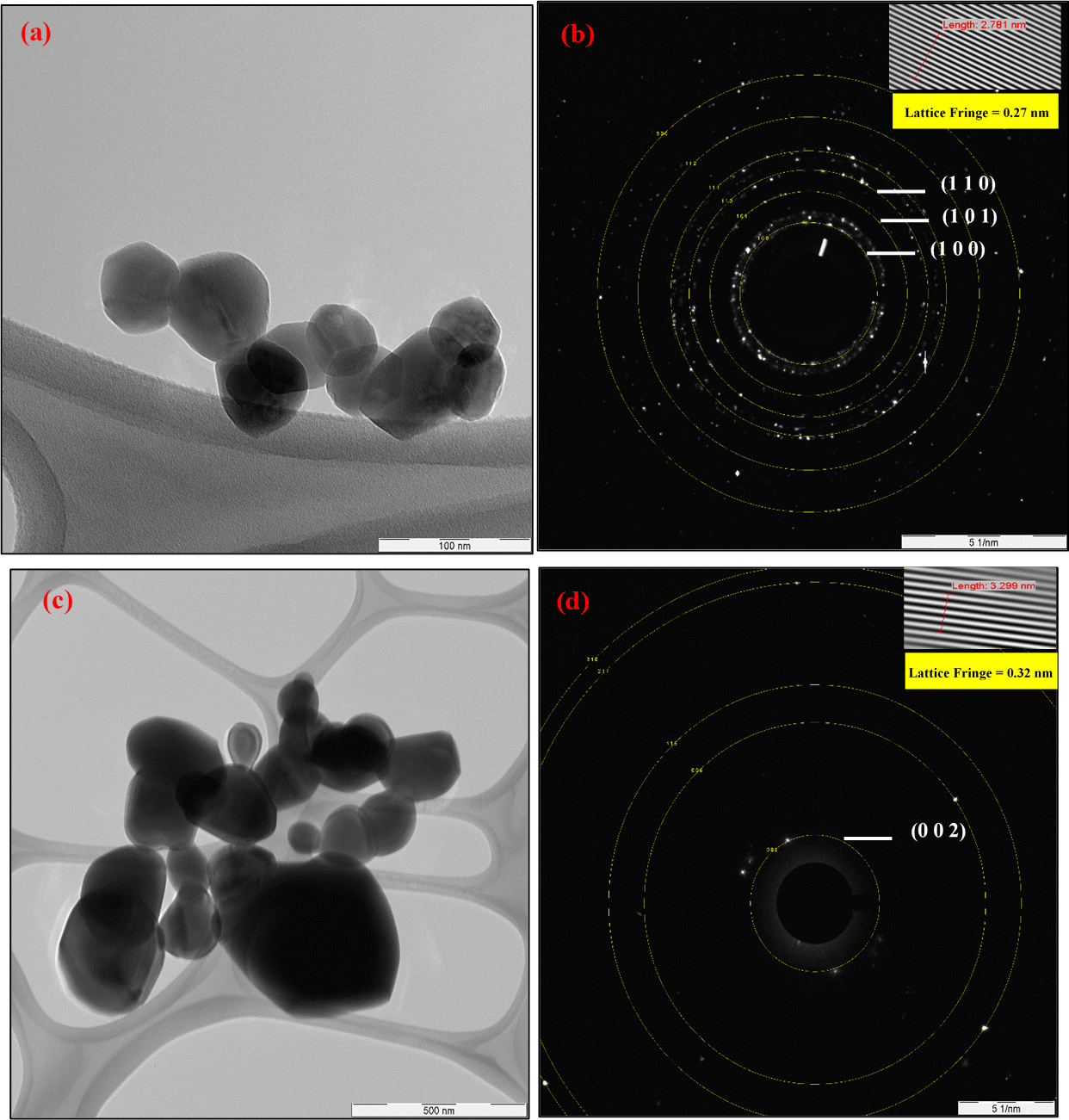

Supplement: S1 Fig — TEM images of ZnO nanoparticles (a, c) calcined at 500°C and 800°C respectively, and their corresponding SAED images (b, d) with inset images of lattice scale fringes. (TIF) [file pone.0244738.s001.tif]

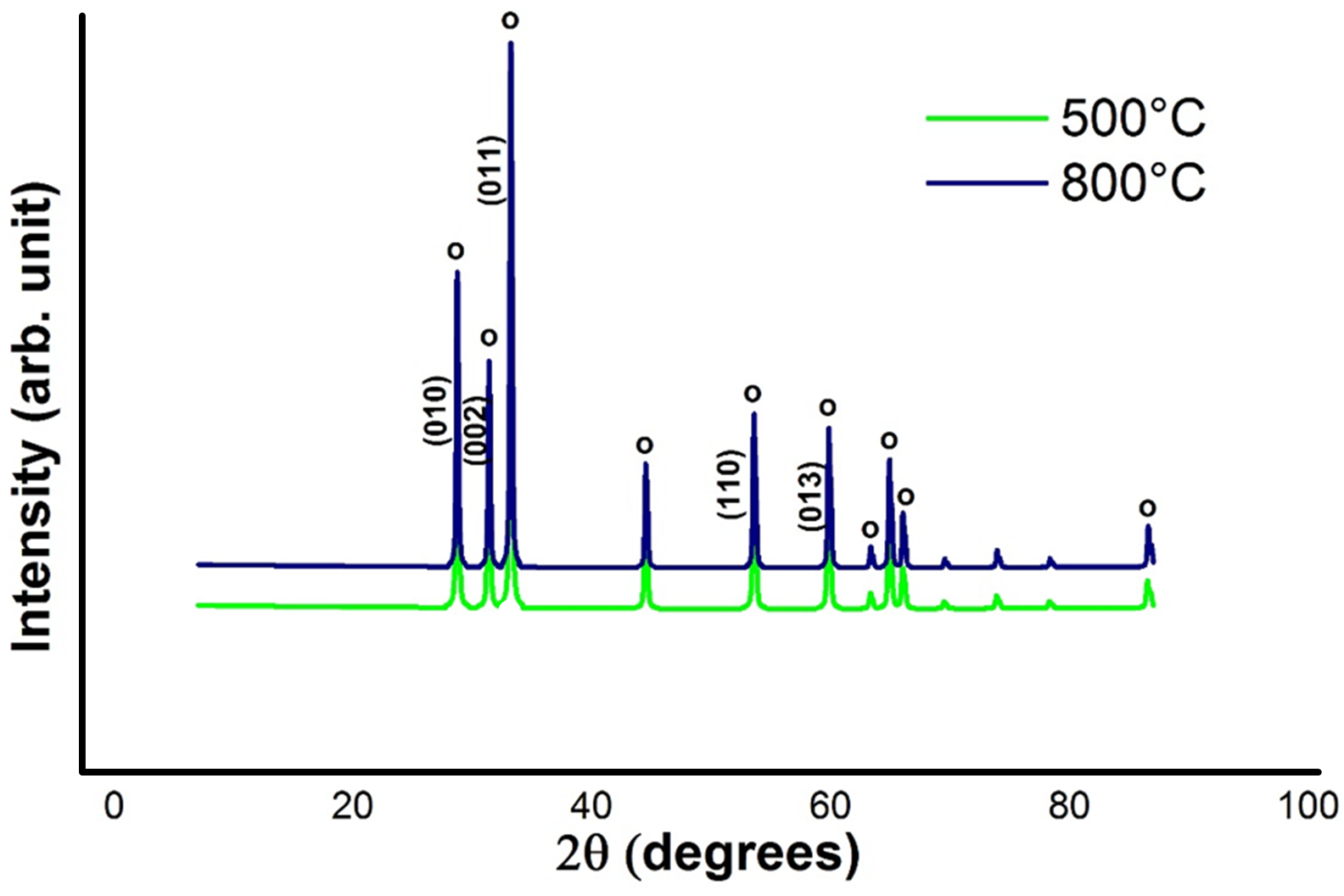

Supplement: S2 Fig — X-ray diffraction patterns of ZnO nanoparticles calcined at 500°C and 800°C. (TIF) [file pone.0244738.s002.tif]

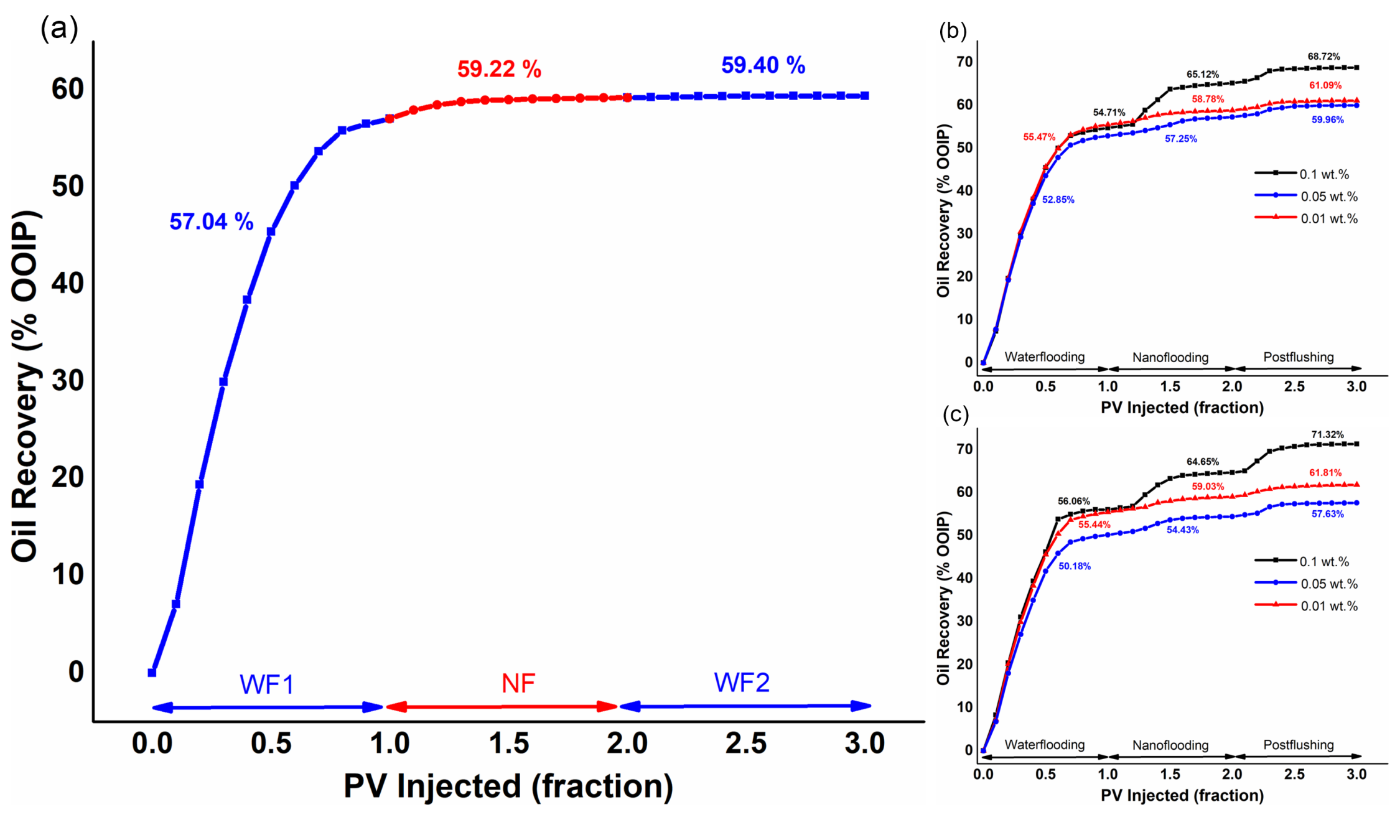

Supplement: S3 Fig — Cumulative oil recovery as a function of injected PV for surfactant flooding of (a) 0.25 wt.% SDBS, and conventional nanofluid flooding of different NPs concentration of (b) ZnO@500 NFs and (c) ZnO@800 NFs. (TIF) [file pone.0244738.s003.tif]

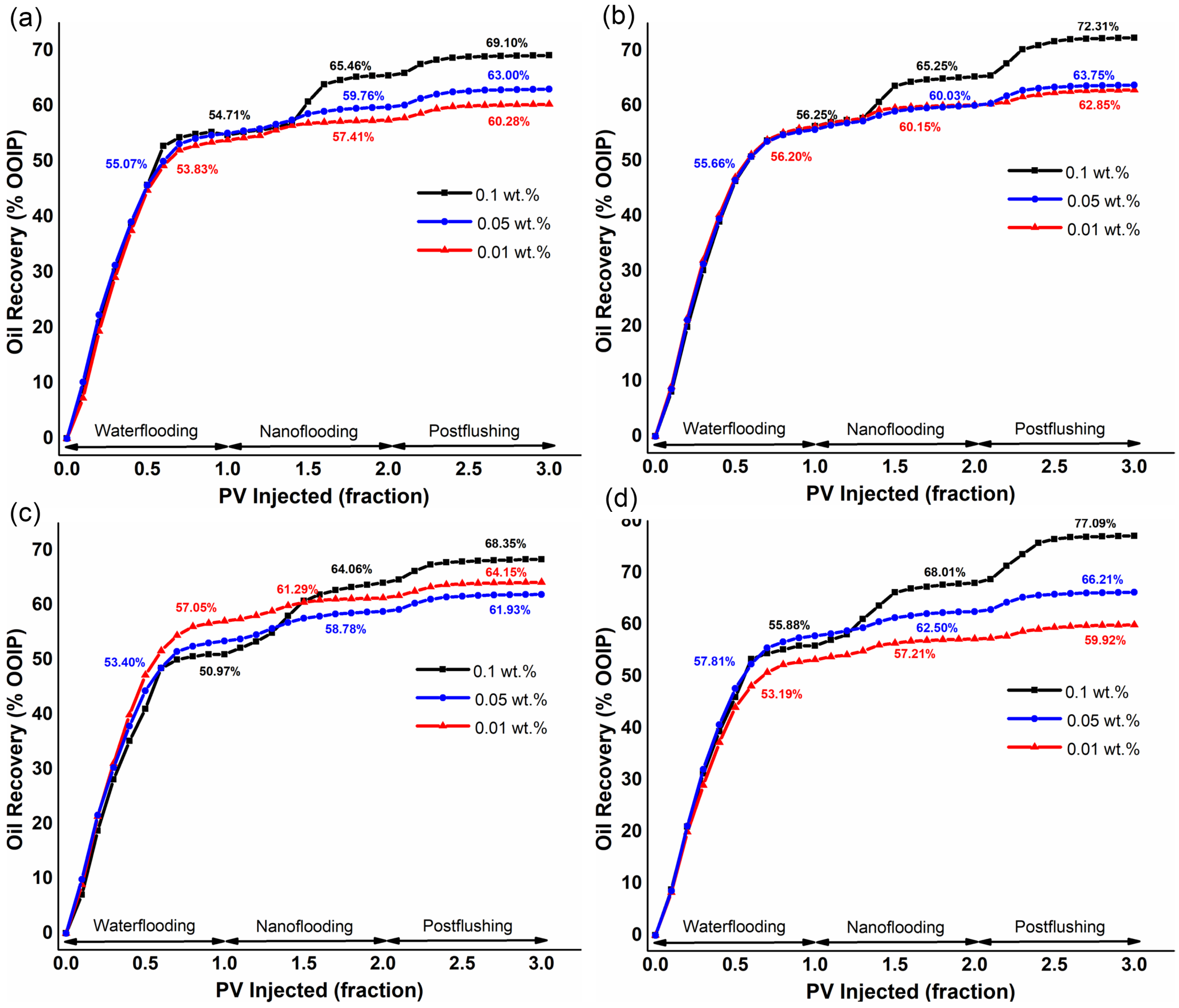

Supplement: S4 Fig — Recovery performance of EM-assisted nanofluid flooding as a function of injected PV for different NPs concentration of (a, c) ZnO@500 NF and (b, d) ZnO@800 NF at an applied frequency of 18.8 and 167 MHz, respectively. (TIF) [file pone.0244738.s004.tif]
